# Epidemiology of Adult Ankle Fractures: 1756 cases identified in Norrbotten County during 2009–2013 and classified according to AO/OTA

**DOI:** 10.1186/s12891-018-2326-x

**Published:** 2018-12-13

**Authors:** Hans Juto, Helena Nilsson, Per Morberg

**Affiliations:** Sunderby Research Unit, Department of Surgical and Perioperative Sciences (orthopaedics), Umeå University, Umeå, Sweden

**Keywords:** Ankle fracture, AO/OTA-classification, Epidemiology, Incidence, Osteoporosis

## Abstract

**Background:**

The ankle fracture is one of the most common fractures, increasing in an ageing population, but not generally seen as an osteoporotic fracture. The aim of this study was to examine the relationship between different AO/OTA classes of ankle fractures, age, sex and type of trauma.

**Methods:**

Ankle fractures, treated at any of the hospitals in Norrbotten County in Sweden between 2009 and 2013, were retrospectively identified and classified according to the AO/OTA-classification system. Information about the trauma mechanism was also obtained.

**Results:**

In Norrbotten County, 1756 ankle fractures in 1735 patients aged 20 years or older were identified. This gave an incidence in the county of 179 per 100,000 person-years. Of these patients, 34.6% were 65 years or older, 58.4% were women and 68.2% of the trauma leading to a fracture was defined as low-energy. In 1.5% of the cases the fractures were open. Incidences of type B fractures increased substantially with age, from 62 (95% CI 50–77) at 30–39 years of age to 158 (95% CI 131–190) in patients older than 80 years of age per 100,000 person-years. Type B fractures showed a slightly higher proportion of low-energy trauma while type C showed a lower mean age and proportion of women.

**Conclusions:**

This study shows an incidence of 179 adult ankle fractures annually per 100,000 persons. More than two thirds of the fractures were caused by a low-energy trauma and ankle fractures are more frequent among females. Females generally have an increased incidence during their life, mainly between the ages of 30 and 60. This is in contrast to men who have more of an even distribution throughout their life. Classification according to AO/OTA reveals some heterogeneity among the classes of ankle fractures in age and gender as well as the energy involved in the trauma.

## Background

Ankle fractures are one of the most common fractures among the adult population. It is the fourth most frequent fracture type after hip, wrist and hand fractures registered in the Swedish Fracture Register and it constitutes approximately every tenth fracture [1]. Similar numbers were found by Court-Brown et al. in the UK [2]. Ankle fractures are also the second most common fracture requiring hospitalization. However, the length of hospitalization decreases year-by-year [3, 4]. The incidence in different studies varies from 71 to 187 per 100,000 person-years [4–9]. It is generally accepted that the incidence of ankle fractures is rising, particularly among the elderly and female population [4–6, 8, 10].

Ankle fractures are commonly classified by the Danis-Weber, the Lauge-Hansen or the AO/OTA classification system [11]. The AO/OTA classification system of ankle fractures can be seen as a development of the Danis-Weber classification with both the height of the fibula fracture taken into account as well as the number of fractured malleoli and comminution of the fibula. The groups of the AO/OTA classification system can also be translated to the Lauge-Hansen system [12].

Ankle fractures are not regarded as the typical fracture associated with osteoporosis. While earlier studies on osteoporosis conclude that fractures of the wrist, humerus, vertebra and hip, for example have a significant relationship with low bone mass, ankle fractures do not [13, 14]. It is also stated that a general increase in age-related incidences is not found among ankle fractures [14, 15]. The majority of ankle fractures are found among patients under the age of 60. [2–4, 7]. In contrast to this, ankle fractures have been shown to increase the risk of a new fracture among postmenopausal women [16]. The epidemiology of ankle fractures is changing and while some earlier studies have reported a male predominance [6, 7] more recent studies now show a significantly higher incidence among females [4, 8, 17]. The age-adjusted incidence of ankle fractures in Finland amongst women over the age of 65 more than doubled between 1970 and 2000. [10]. The majority of studies show that there is a rise in incidence among women to the age of about 60 to 70 and thereafter it levels out or even declines. [3–7, 17–20]. Multimalleolar ankle fractures though, appear to increase with age amongst female patients [4, 5].

While ankle fractures are common, there are reasons to continue studying its epidemiology to better understand the fracture. The aim of this study is to examine the incidence of ankle fractures in Norrbotten County as well as the relationship between AO/OTA-classes of ankle fractures with age, gender and low-energy trauma.

## Methods

### Identification of fractures

Norrbotten County is the most northerly and sparsely-populated county in Sweden. Ankle fractures are treated at five emergency departments and six rural health care centres. The operative treatment of fractures is conducted at one main trauma hospital (Level II Trauma Centre) and another smaller hospital (Level III Trauma Centre). All hospitals and departments share the same computerized medical record system (VAS), where documentation and X-rays can be obtained. When a patient is treated in a hospital in Sweden there is a mandatory registration of the ICD-10 diagnosis linked to the appointment. Therefore, coverage of ankle fractures through VAS should be close to 100%.

Ankle fractures were identified in patients, 20 years of age or older, through a search in VAS using ICD-10 codes for open- and closed-ankle fractures (S82.50-S82.81). The ankle sprain injury has its individual ICD-10 code (S93.4) and was not included in the search.

To obtain information about the trauma mechanism, information in the referral text was mainly used, complemented in some cases with information from medical records. Low-energy trauma was defined in this study as a fall from a standing height or less, with an eventual physical activity not more than walking. The remaining fractures, including sports injuries and falls down stairs, were defined as non-low-energy trauma. All X-rays of the ankle fractures were extracted from the system, anonymized and numbered. The side (right or left) and date of X-ray was indicated. Patients who fractured their ankles more than once during the period of the study, or patients with a bilateral ankle fracture were identified similarly. Patients who had an ankle fracture diagnosis recorded in different years were checked in the medical record, to lower the risk of not detecting multiple injuries.

Due to the relatively high degree of injury misclassification, the search in VAS was extended by using ICD-10 codes for distal tibia fractures and isolated fibula shaft fractures. (S82.30–82.41). Before obtaining X-rays of these cases, an assessment was first conducted from the referral and medical records, as well as the X-rays, in order to detect if there were cases of ankle fractures misclassified as distal tibia or fibula shaft fractures.

Information about the patients’ place of residence is continuously updated in VAS from the governmental residential information database (SPAR), and patients not residing in the county were identified by information in this database and in the medical records. Patients residing in the county, but injured and treated elsewhere, but followed up at home could also be identified.

### Classification

The AO/OTA classification system for ankle fractures consists of three classes, nine groups and 27 subgroups. The A11 subgroup is defined as only a sprain and was therefore omitted. In addition to the subgroups, the cases could be defined as “no fracture”, “other fracture than ankle” or “ankle fracture but not possible to classify”.

Three different observers (HJ, HN and PM) classified the fractures into four levels according to the AO/OTA-system. The instructions at the AO foundation online surgery reference were used as guidelines for the classification [12]. The assessments were made by each observer independently, at their own pace and without access to each other’s results. If there was agreement in at least two out of three assessments, the classification was considered final. In cases where there was total disagreement, the fracture was discussed at a final meeting and a majority decision finalized the classification.

### Population

The mean adult population in the county during the study period was 196,351 people. The mean age of the total population in the county during the study was 43.3 years. Of the adult population, 49.5% were female [21].

### Statistics

Descriptive Statistics in SPSS Statistics version 24 was used to calculate the number in each group, e.g. the number of females over 65 with a B2 fracture. The confidence interval of 95% was calculated in Openepi.com, using the Wilson method for binomial proportion and Mid-P exact test for incidence.

## Results

### Identified fractures

Between 2009 and 2013, 1851 unique patients aged 20 or older with ICD-10 S82.50-S82.81 (open or closed ankle fracture) were identified. Out of this group of patients, 145 resided outside of the county and were therefore excluded. Another 125 cases were classified from the X-rays, as non-ankle fractures and were also excluded. Thirty-eight of them were patients with diverse types of tibia fractures that had been incorrectly diagnosed as ankle fractures. Fifty were classified as either an avulsion fracture or as a sprain and accordingly excluded. Within the five-year time period of the study, seven patients had fractured both ankles in the same injury. In eleven cases the patients had sustained another (in one case two more) ankle fractures, leading to an additional 19 fractures.

**Fig. 1:**
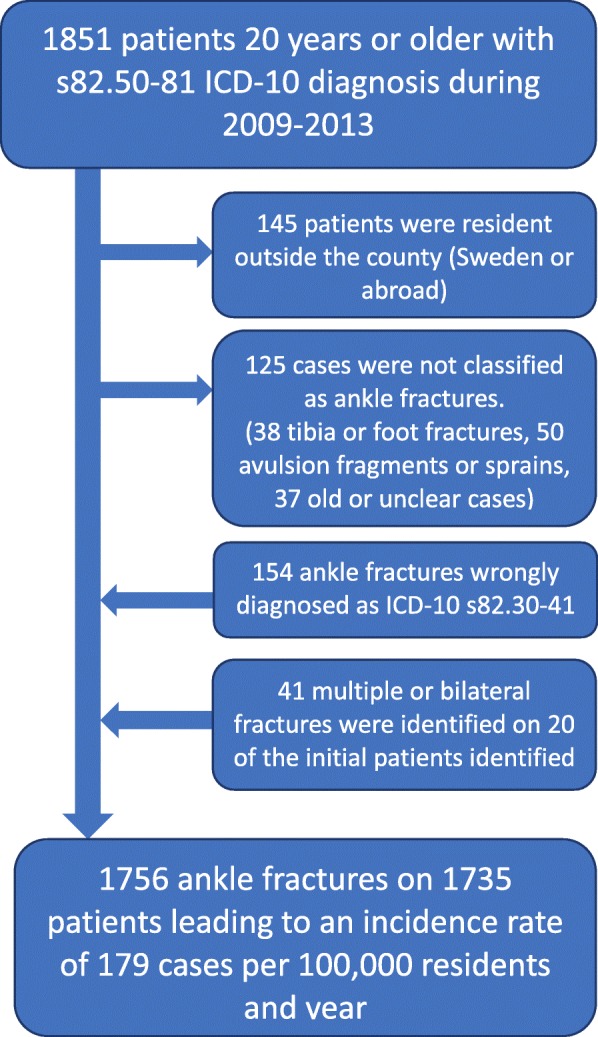
Flow-chart identification of ankle fracture in computerized medical documentation system

An additional 154 unique patients with 156 ankle fractures within the study period were identified as incorrectly diagnosed with the ICD-10 code S82.30–82.41 (distal tibia or fibula shaft). This resulted in 1756 validated ankle fractures in 1735 patients, 20 years of age or older and resident in the county (Fig 1).

### Classification

All the ankle fractures were classified according to the AO/OTA-system by type, group and subgroup. Out of the initial cases (including other fractures and ankle fractures of non-residents, for example) there was complete agreement in the classification of the AO/OTA subgroup level between the three observers in 56% of the cases. In another 36% there were two observers against one and in 9% of the case there was total disagreement and those cases needed to be discussed at a meeting. The interobserver agreement amongst the raters was between 90 and 91% for AO/OTA type level, 75–81% for AO/OTA group and 65–75% for AO/OTA subgroup level.

Regarding ankle fractures, 19.8% were classified as a type A fracture, 66.2% as a type B and 10.6% as a type C fracture. The distribution between the subgroups was uneven and the five largest subgroups constituted 58.8% of all fractures. B11 was by far the largest subgroup with 484 (27.6%) cases while some subgroups like A31-A33, C12 and C32 included only very few cases (Table 1).

**Table 1:** Characteristics on 1756 cases of ankle fracture. Number and proportion of the different classes as well as proportion of elderly patients, women and low energy trauma. 95% CI was calculated using Wilson method

AO class	Number, n (%)	> 65 years	Women	Low energy
A	347 (19.8)	35.2 (30.3–40.3)	60.2 (55.0–65.2)	58.8 (53.6–63.9)
A1	243 (13.8)	36.6 (30.8–42.9)	67.9 (61.8–73.5)	64.9 (58.7–70.6)
A12	136 (7.7)	36.8 (29.1–45.1)	66.9 (58.6–74.2)	62.5 (54.1–70.2)
A13	107 (6.1)	36.4 (27.9–45.9)	69.2 (59.9–77.1)	67.9 (58.5–76.1)
A2	91 (5.2)	31.9 (23.2–42.0)	40.0 (30.5–50.3)	43.3 (33.6–53.6)
A21	71 (4.0)	25.4 (16.7–36.6)	31.4 (21.8–43.0)	37.1 (26.8–48.9)
A22	11 (0.6)	63.6 (35.4–84.8)	72.7 (43.4–90.3)	54.5 (28.0–78.7)
A23	9 (0.5)	44.4 (18.9–73.3)	66.7 (35.4–87.9)	77.8 (45.3–93.7)
A3	13 (0.7)	30.8 (12.7–57.6)	61.5 (35.5–82.3)	53.8 (29.1–76.8)
A31	7 (0.4)	14.3 (2.6–51.3)	57.1 (25.1–84.2)	57.1 (25.1–84.2)
A32	2 (0.1)	50.0 (9.5–90.6)	50.0 (9.5–90.6)	100.0 (34.2–100.0)
A33	4 (0.2)	50.0 (15.0–85.0)	75.0 (30.7–95.4)	25.0 (4.6–69.9)
B	1162 (66.2)	36.7 (33.9–39.5)	60.1 (57.2–62.9)	72.0 (69.4–74.5)
B1	649 (37.0)	36.5 (32.9–40.3)	53.0 (49.2–56.8)	71.6 (68.1–75.0)
B11	484 (27.6)	34.7 (30.6–39.1)	53.9 (49.5–58.3)	71.1 (66.9–74.9)
B12	145 (8.3)	42.8 (35.0–50.9)	49.0 (41.0–57.0)	73.8 (66.1–80.3)
B13	20 (1.1)	35.0 (18.1–56.7)	60.0 (38.7–78.1)	70.0 (48.1–85.5)
B2	221 (12.6)	35.7 (29.7–42.3)	59.7 (53.2–66.0)	72.4 (66.1–77.9)
B21	96 (5.5)	26.0 (18.3–35.6)	46.9 (37.2–56.8)	74.0 (64.4–81.7)
B22	111 (6.3)	45.0 (36.1–54.3)	69.4 (60.3–77.2)	72.1 (63.1–79.6)
B23	14 (0.8)	28.6 (11.7–54.7)	71.4 (45.3–88.3)	64.3 (38.8–83.7)
B3	292 (16.6)	37.7 (32.3–43.3)	76.0 (70.8–82.4)	72.6 (67.2–77.4)
B31	100 (5.7)	32.0 (23.7–41.7)	65.0 (55.3–73.6)	72.0 (62.5–79.9)
B32	156 (8.9)	42.3 (34.8–50.2)	80.8 (73.9–86.2)	71.2 (63.6–77.7)
B33	36 (2.1)	33.3 (25.6–55.3)	86.1 (71.3–93.9)	80.6 (65.0–90.3)
C	189 (10.6)	25.4 (19.6–32.1)	48.7 (41.7–55.8)	62.4 (55.4–69.0)
C1	95 (5.4)	29.5 (21.3–39.3)	56.8 (46.8–66.3)	64.2 (54.2–73.1)
C11	61 (3.5)	29.5 (19.6–41,9)	54.1 (41.7–66.0)	70.5 (58.1–80.4)
C12	5 (0.3)	60.0 (23.1–88.2)	60.0 (23.1–88.2)	60.0 (23.1–88.2)
C13	29 (1.7)	24.1 (12.2–42.1)	62.1 (44.0–77.3)	51.7 (34.4–68.6)
C2	59 (3.4)	18.6 (10.7–30.4)	47.5 (35.3–60.0)	59.3 (46.6–70.9)
C21	22 (1.3)	9.1 (2.5–27.8)	31.8 (16.4–52.7)	59.1 (38.7–76.7)
C22	8 (0.5)	37.5 (13.7–69.4)	50.0 (21.5–78.5)	37.5 (13.7–69.4)
C23	29 (1.7)	20.7 (9.8–38.4)	58.6 (40.7–74.5)	65.5 (47.6–80.1)
C3	35 (2.0)	25.7 (14.1–42.1)	28.6 (16.3–45.1)	62.9 (46.3–76.8)
C31	18 (1.0)	27.8 (12.5–50.9)	22.2 (8.5–43.3)	61.1 (38.6–79.7)
C32	3 (0.2)	33.3 (6.15–79.2)	0.0 (0.0–56.2)	0.0 (0.0–56.2)
C33	14 (0.8)	21.4 (7.6–47.6)	42.9 (21.4–67.4)	78.6 (52.4–92.4)
Unclassified	58 (3.3)	20.7 (12.3–32.8)	43.6 (31.4–56.7)	67.3 (53.8–78.5)
Total	1756 (100.0)	34.6 (32.4–36.8)	58.4 (56.1–60.7)	68.2 (66.0–70.4)

### Incidence

**Fig. 2:**
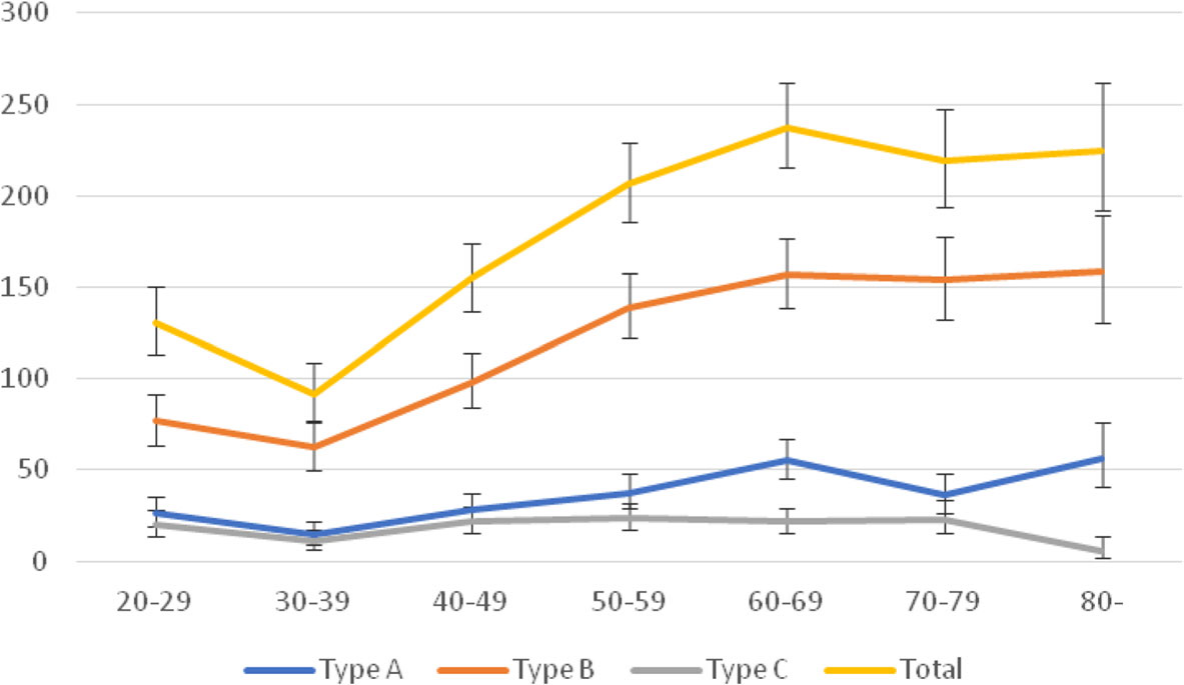
Incidence per 100,000 person and year according to AO/OTA type and age. Ninety five percent CI was calculated using Mid-P exact test

The incidence of adult ankle fractures in Norrbotten County was 179 cases per 100,000 person-years during the study period. The lowest incidence was seen between 30 and 39 years of age. There was a rise in the incidence peaking at 60–69 years. A plateau with a small but non-significant decrease was thereafter reached. The incidence increased with age from 92 (95% CI 77–109) cases per 100,000 person-years at 30–39 years of age to 238 (95% CI 216–262) cases per 100,000 person-years at 60–69 years of age (Fig. 2). When adjusting the incidence for the age distribution of Sweden, in its entirety, it decreases slightly to 172 cases per 100,000 person-years.

**Fig. 3:**
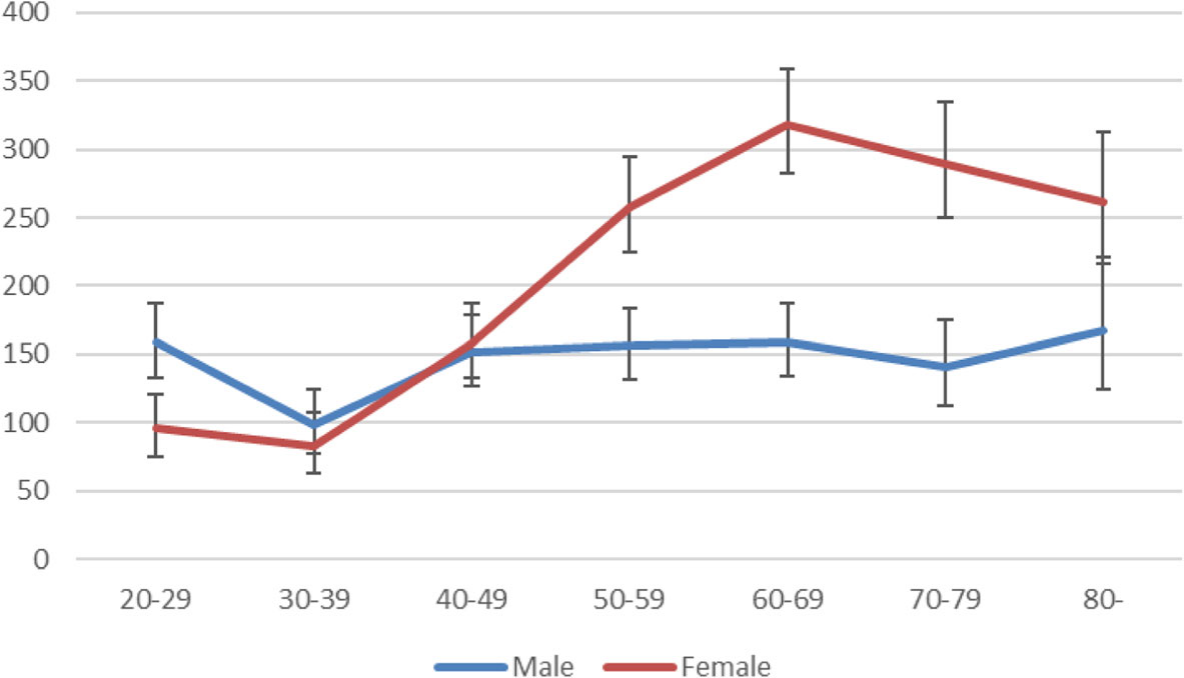
Incidence per 100,000 persons and year according to gender and age. Ninety five percent CI was calculated using Mid-P exact test

Men had a much more even distributional incidence throughout their lives than females, with only a dip between 30 and 39 years of age. Among females, the incidence increased from 83 (95% CI 63–108) per 100,000 person-years at 30–39 to 319 (95% CI 283–359) at 60–69 years (Fig. 3).

The incidence of type B fractures increased substantially with age and had a similar pattern as ankle fractures. From 62 (95% CI 50–77) at 30–39 years to 158 (95% CI 131–190) at 80 years of age and older per 100,000 person-years. The incidence of type A fractures also increased similarly but not as markedly. From 14 (95% CI 9–22) at 30–39 to 56 (95% CI 45–68) per 100,000 person-years at 60–69 years. Type C fractures had a quite even incidence but with a significantly lower incidence at 30–39 and 80 years of age or older (Fig. 2).

### Characteristics of ankle fractures

The mean age of patients in the study with an ankle fracture was 56.3 (range 20–101) years and 34.6% were 65 years of age or older. The percentage of female patients was 58.4 and 68.2% of the trauma leading to the fracture was defined as low-energy. In 1.5% of the cases the fracture was open.

**Fig. 4:**
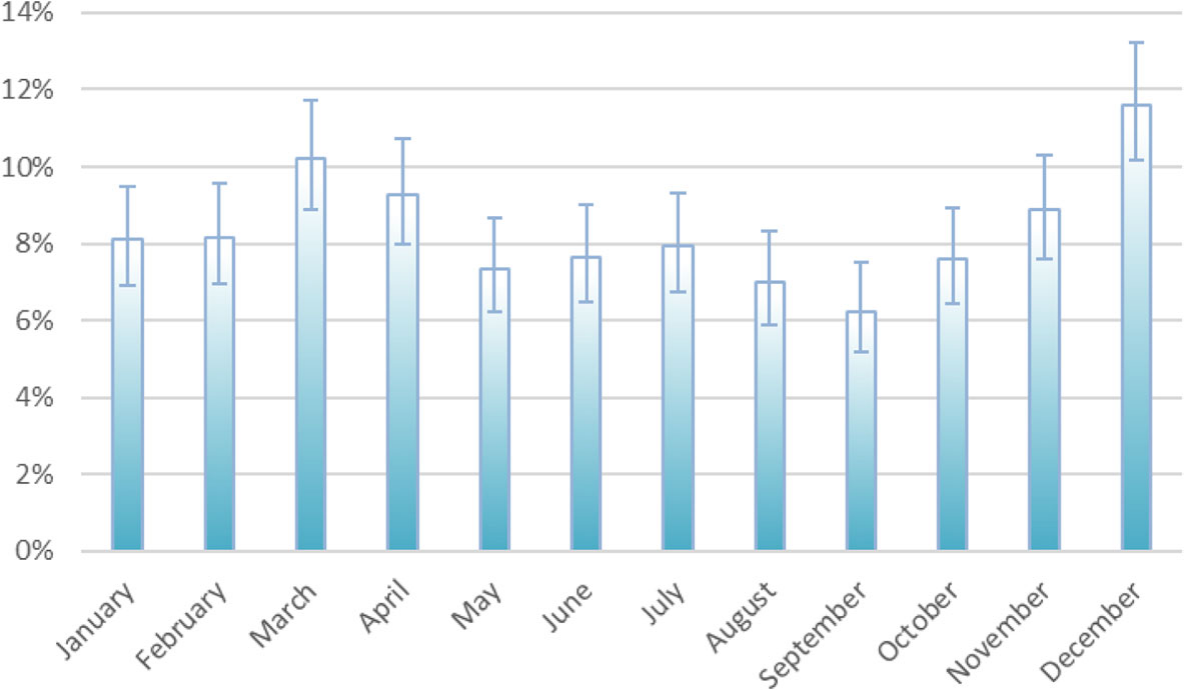
Distribution of fractures throughout the year. Ninety five percent CI was calculated using Wilson method

**Fig. 5:**
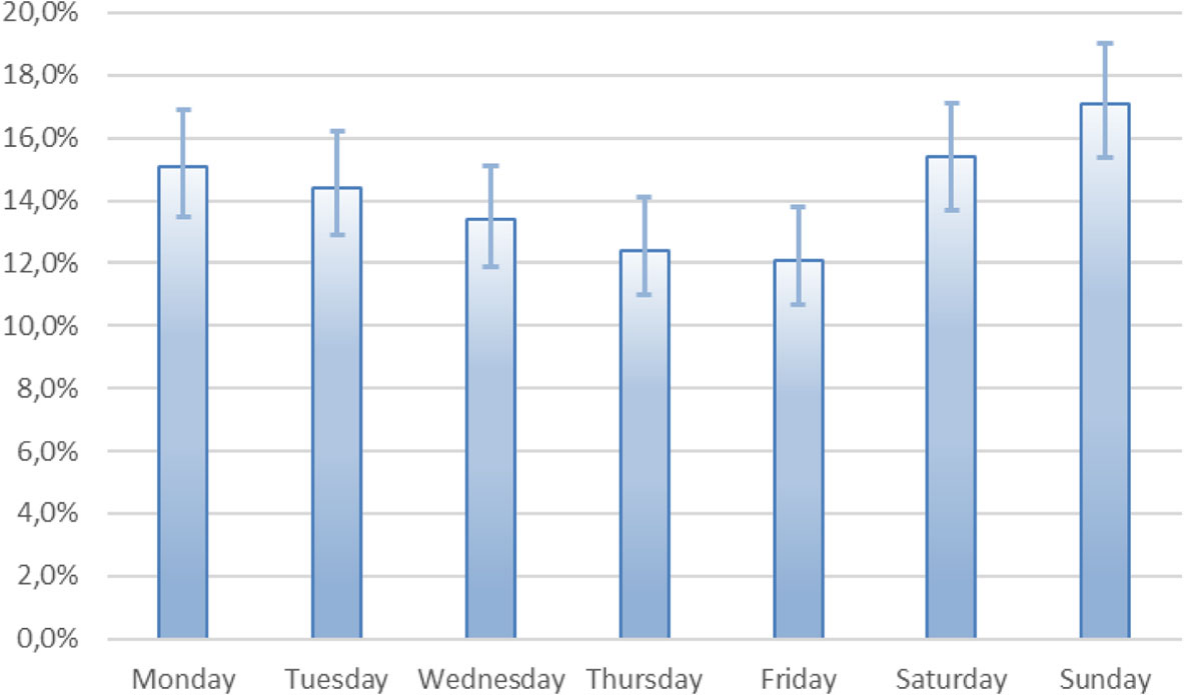
Distribution of fractures throughout the week. Ninety five percent CI was calculated using Wilson method

There were more fractures of the right ankle, 53.3% compared to 46.7%. There was also a difference in the frequency of ankle fractures during the year, with three prominent peaks, in December, March and July, while the lowest incidence was in September (Fig. 4). More ankle fractures were observed during the weekend (Fig. 5).

### Characteristics of subgroups

Type A, the infra-syndesmotic fractures, showed a smaller number of low-energy trauma than ankle fractures as a whole, but showed no difference in frequency for women or the elderly. Within the subgroups of type A fractures, a somewhat heterogeneous pattern is observed. The isolated medial malleolus fracture, A21, occurred significantly less frequently among females and after less low-energy trauma (Table 1).

The trans-syndesmotic type B fractures, which constituted two thirds of the total number of ankle fractures, had a slightly higher and significant number of low-energy fractures than type A and C fractures. The multi-malleolar type B fractures like B22, B32 and B33 has a somewhat higher frequency of females or/and elderly than unimalleolar B21fractures (Table 1).

Type C or the supra-syndesmotic fracture showed a significantly lower number in elderly and female patients than ankle fractures as a whole, and a lower amount of low-energy trauma than type B fractures (Table 1). Eight supra-syndesmotic fractures were included in the type C classification, even though they did not completely fulfil the conditions as they were equivalent to C13 and C23, but without a medial malleolus fracture.

### Unclassified cases

Fifty-eight cases could not be classified according to the AO/OTA system. In 24 cases, pre-treatment X-rays were missing, often because the patients were surgically treated at hospitals outside Norrbotten County. In another 26 cases they only consisted of an isolated posterior malleolus fracture. Three of these cases were caused by axial force, five by dorsal flexion, 13 by twist and in five cases the injury could not be classified.

## Discussion

This study shows an incidence of 179 ankle fractures per 100,000 person-years in the county. In comparison to both earlier and similar studies, this study shows a slightly higher incidence. The incidence of ankle fractures has been reported as between 71 and 187 per 100,000 person-years. [4–9]. We see a few reasons for this high incidence in Norrbotten County. Different presuppositions and methodology does, of course, make it difficult to make a completely identical comparison.

A major strength of this study is that we believe we have found almost every possible ankle fracture in the county during the period of study and validated them by examining and classifying every case. However, the strength of the study is also in a way its weakness. The population is relatively small, in a limited area in the far north of Sweden. One can question whether it is possible to generalize the results. Another weakness is that it is a quite low interobserver agreement of the subgroup level of the AO/OTA classification of ankle fracture that can affect the results in the smaller subgroups (e.g. A32 and A33). Even though we have gone to great lengths to find all fractures there may be scenarios where cases have escaped our search. One would be if a patient injured outside the county and did not return within the period of the cast immobilisation.

Elsoe et al. recently published a study on ankle fracture epidemiology in Denmark, reporting an incidence of 169 per 100,000 person-years [8]. In Norrbotten County the mean age of the population is about 2.5 years higher than Sweden’s general population [21]. Adjusted for this age difference, the incidence equates to 172 adult ankle fractures per 100,000 person-years. Our results are thereby very similar to those of Elsoe et al. and it seems that this is the true incidence for the period of the study and the region. We can expect similar countries to have a similar incidence.

There is general agreement that the incidence of ankle fractures increases with time, due to an ageing population [4–6, 8, 10]. We agree with this and believe it to be the main reason for the relatively high incidence in our population. The population in Norrbotten County is ageing relatively quickly, the mean age increasing from 40.5 years in 1998 to 43.7 years in 2015 [21]. Another factor that could explain a higher incidence is the long winter season in the county. The incidence increases markedly at the beginning and the end of the winter, when the average temperature is around zero degrees Celsius, resulting in icy and slippery roads and pavements. Our data shows an increase in incidence during the weekend, implying that a general increase in activity, as expected, is also a factor.

One third of the adult ankle fracture patients in the study were 65 years or older. We also observed an increase in incidence from the age of 30–39 to the age of 60–69 and this age group has the highest incidence. This rise in incidence is almost totally caused by the rise among females of nearly four times while men have a more even spread throughout their lives. Elsoe et al. show a similar pattern of incidence but report a decreasing trend among men [8]. We chose not to present the age span over 90 years of age separately, as this group is very small and hence the confidence interval is large.

There is controversy regarding ankle fractures. Should it be characterised as an osteoporotic fracture or not [2, 5, 8, 13, 14, 22–30]? Since a fracture is one of the strongest risk factors for another fracture this is still an important question [31]. A high frequency among women, low-energy trauma as a cause and the incidence increasing with age is seen in osteoporotic fractures. [23, 24]. The proportion of low-energy trauma, as a cause of fractures, was two-thirds in this study. Thur et al. report similar proportions, but in comparison to earlier studies it is slightly higher [4, 6, 9]. This is probably an additional result of an ageing population.

The increase of incidence with age is shown in a number of earlier studies where the highest incidence in females occurs after the age of 65 [4, 6, 8]. Furthermore, a study published recently show an association between lower bone mineral density and ankle fractures [30].

It is difficult to explain the pattern with the rising incidence among women both by age and time, without explaining it as being due to loss of strength in the bone. However, when using a definition of a fragile fracture due to low-energy trauma in an elderly person, they only constitute around one out of four fractures [8, 10]. With a prominent component of osteoporosis as cause of the fracture, this would contradict the plateau in incidence reached after 60 years of age.

We show some differences between the different classes of ankle fracture in the amount of low-energy trauma, gender and age. At one end of the spectrum are the multimalleolar trans-syndesmotic ankle fractures corresponding to B22–3 and B3 representing about every fifth ankle fracture. These occur in female patients in about three-quarters of cases and are caused by low-energy trauma in 70% of cases. It is difficult not to see bone fragility, at least partly, as a cause of this.

At the other end of the spectrum we found type A21 fractures and C fractures. A21 is equivalent to an isolated medial malleolus fracture and shows clear signs of being a non-osteoporotic fracture. It is the one subgroup in our population that stands out and has a low proportion of elderly people; caused in a majority of the cases by a non-low-energy trauma and the patients are mainly men. This is also in agreement with earlier studies [4, 6, 32].

The supra syndesmotic type C ankle fracture has been described as less of an osteoporotic fracture with a lower mean age and a lower frequency of low-energy trauma [6, 9]. Sakaki et al. demonstrate in their study in São Paulo of surgically-treated ankle fractures with a high amount of high-energy trauma that type C fractures compose about 37% of the fractures. More than half of them were high-energy traffic accidents [33]. Briet et al. show in a similar study that all Lauge-Hansen classes except supination external rotation fractures (SER) is, in a majority of the cases, caused by high energy trauma. [32]. In our population, type C fractures show a similar pattern with fewer elderly females and low-energy trauma. However, low-energy trauma is still the cause in over 60% of type C fractures and still one out of four patients are 65 years of age and older.

Court-Brown et al. present a theory where they give medial, lateral, bimalleolar, trimalleolar and supra-syndesmotic ankle fractures different incidence patterns [2]. Even though we do not see this somewhat simplified pattern, we noticed a tendency that supports the theory of an increase with age in lateral malleolus and bimalleolar and trimalleolär ankle fractures, but not in the same way in medial malleolar and supra-syndesmotic ankle fractures.

Compared with Thur et al., we see a lot of similarities in the incidence pattern, except for two differences. One is the decrease in incidence of bimalleolar and trimalleolar fractures after 70 years of age in their study. This can be due to the fact that type C is included in that category. The other difference is that the incidence of lateral malleolar fractures is very low. Our incidence of these is about 2–3 times higher, probably because of the limitation of only including inpatients [4].

## Conclusion

This study shows an incidence of 179 adult ankle fractures annually per 100,000 persons. More than two thirds of the fractures were caused by a low-energy trauma and ankle fractures are more frequent among females. Females generally have an increased incidence during their life, mainly between the ages of 30 and 60. This is in contrast to men who have more of an even distribution throughout their life. Classification according to AO/OTA reveals some heterogeneity among the classes of ankle fractures in age and gender as well as the energy involved in the trauma.

## Abbreviations

AO: Arbeitsgemeinschaft für osteosynthesefragen
ICD-10: International Statistical Classification of Diseases and Related Health Problems - Tenth Revision
OTA: American Orthopaedic Trauma Association
VAS: Vårdadministrativt System (Digital medical record and administrative system)
